# Tears and cheers: A narrative inquiry of a doctoral student’s resilience in study abroad

**DOI:** 10.3389/fpsyg.2022.1071674

**Published:** 2022-12-13

**Authors:** Xinxin Wu

**Affiliations:** School of Foreign Studies, Jiangnan University, Wuxi, China

**Keywords:** doctoral student, Ph.D. student, resilience, study abroad, narrative inquiry

## Abstract

The existing literature has revealed many issues related to Ph.D. students’ wellbeing, such as anxiety and stress, which are likely to cause Ph.D. student attrition or dropout. As one of the key coping strategies against psychological burnout, resilience has received increasing attention among various teacher groups. However, it still lacks a systematic understanding of students, in particular overseas Ph.D. students. This article explores doctoral students’ resilience through a narrative inquiry into the resilience-building process of a Ph.D. student in language and linguistics, Hongxia, in the United Kingdom. Drawing on the research methods and results of resilience from teachers, this study examines Hongxia’s lived experience as a Ph.D. student throughout her 3 years of learning and research and uncovers the dynamic and multifaceted process of resilience building as the interplay between Ph.D. students’ agency, peers, supervisors, academics, families, and friends. The current research supports the value of narrative inquiry, in particular critical story analysis, as a tool for studying the resilience-building processes in Ph.D. students during their candidature. It also hopes to provide insight for administrators, supervisors, and other related stakeholders on their intervention to support and facilitate the research journey of doctoral students.

## 1 Introduction

Ph.D. or doctoral students are a special student group in higher education institutions who are confronted with an exceptionally long and difficult journey to the completion of their degree (Smith et al., 2006) as opposed to other postgraduate or undergraduate students. Research has revealed that this group of students is often associated with a poor status of wellbeing (Kismihók et al., 2022). Specifically, Ph.D. students normally need to undertake a range of demanding academic tasks such as finishing required courses, passing the candidate examination, and writing a thesis in order to be awarded a doctoral degree. Besides such intensive work, Ph.D. students often need to attend to other life responsibilities, such as family commitments, job requirements, and financing issues (Smith et al., 2006; Moate et al., 2019). Struggling with these demanding requirements often brings about high levels of stress and negative emotions among Ph.D. students, posing severe challenges to their wellbeing (Kurtz-Costes et al., 2006; Stubb et al., 2011). The poor status of Ph.D. students’ wellbeing, such as stress and anxiety, is claimed to be the typical reason for their high attrition rate (Nettles and Millett, 2006; Jiranek, 2010; Stubb et al., 2011). Such dropout of Ph.D. students often causes loss to higher education institutions, such as facility allocations, scholarship, supervisory time, and high-quality research outputs for universities (Dhirasasna et al., 2021).

The aforementioned condition enlightened us that it is of paramount importance to figure out the factor influencing Ph.D. students’ wellbeing. As a psychological construct, wellbeing has been widely used to refer to “the state of complete physical, mental, and social wellbeing and not merely the absence of disease or infirmity” (World Health Organization [WHO], 2022). Extant studies have explored a diverse range of factors related to Ph.D. students’ wellbeing (Schmidt and Hansson, 2018), such as workload and control (Mackie and Bates, 2019), level of support (Sverdlik et al., 2018; Sverdlik and Hall, 2020), supervisory relationship (Barry et al., 2018), financial concerns (Metcalfe et al., 2018), research progress (Schmidt and Hansson, 2018), and role conflicts (Guthrie et al., 2018; Yusuf et al., 2020). Other researchers have explored the impact of some demographic factors, such as race and gender, on the wellbeing of Ph.D. students (Paustian-Underdahl et al., 2017; Corvino et al., 2022). However, few studies to date have investigated how individual personality characteristics might affect the wellbeing of Ph.D. students (Perepiczka and Balkin, 2010; Moate et al., 2019). This is surprising, given the vital role of personality characteristics in influencing individuals’ perception of experiences in life (Ghorpade et al., 2007). As one important personality construct, resilience of Ph.D. students warrants our special attention as it has been manifested to be instrumental in the performance and wellbeing of different populations, such as teachers (Johnson et al., 2014; Proietti Ergün and Dewaele, 2021; Chu and Liu, 2022) and general learners (Rosenbaum and Weatherford, 2017; Cleary et al., 2018). The overarching goal of this research is to explore the resilience-building process and its influencing factors through a narrative case study of a Ph.D. student during her candidature in a study abroad context.

## 2 A brief overview of research on resilience

As a construct originated in psychology to solve children’s misconducts such as mental disorders due to severe hazards (Rutter, 1999; Fredrickson, 2004), resilience has also aroused an upsurge of scholarly attention in the field of positive psychology in recent years (Le Cornu, 2009; Gu and Day, 2013; Liu and Chu, 2022; Meierdirk and Fleischer, 2022). However, no consensus has been reached in terms of its exact definition. Overall, the notion of resilience has been conceptualized from an individual’s innate attribute to cope with adversity or threat to “a dynamic process of positive adaptation whereby individuals mobilize internal and external resources” (Lu and Zhu, 2022, p. 3). Based on a critical overview of empirical research findings from a wide variety of disciplines, Gu and Day (2013) summarized three shared considerations in the way resilience is conceptualized: its presupposition of the presence of a threat to the *status quo*, its learned and acquired rather than innate and fixed nature, and its personal characteristics and relationships with the social environment where the individual works and lives. In a word, recent studies are inclined to see resilience as a developmental notion co-constructed by the individual and contexts, rather than an innate or static state.

Furthermore, Richardson et al. (1990) proposed a three-stage model of the resilience-building process in health profession. In the first place, an individual’s organized state of life was broken by particular challenges. Second, the individual struggled against these challenges to reorganize life and regain equilibrium from traumatic experiences. Finally, the individual became more adept at coping strategies to challenges. This model revealed the dynamic nature of the resilience-building process, which is in line with the aforementioned developmental nature of resilience. An investigation of the experience of a doctoral student in a cross-cultural context in this research is informed by this three-stage model of resilience building and the growing literature on the significance of resilience in many aspects of our lives ranging from teacher wellbeing (Hascher et al., 2021) and career enjoyment (Proietti Ergün and Dewaele, 2021) to learner motivation and language learning proficiency (Kim and Kim, 2017; Kim et al., 2019).

In relation to the empirical research on resilience, a majority of studies conducted to date have been focused on teacher resilience (Beltman et al., 2011; Ainsworth and Oldfield, 2019). They include the exploration of relevant theories and theorization of teacher resilience (Ebersoehn, 2014; Mansfield et al., 2016; Drew and Sosnowski, 2019; Hascher et al., 2021), the significance of resilience for teachers (Gu and Day, 2007; Clara, 2017; Li and Lv, 2022), ways of fostering and sustaining teacher resilience (Wang, 2021; Kowitarttawatee and Limphaibool, 2022), and the investigation into resilient features of teachers from a range of sociocultural contexts and educational levels (Day and Hong, 2016; Peixoto et al., 2020; Gratacós et al., 2021; Xue, 2021; Chu and Liu, 2022; Gan et al., 2022; Wang and Lo, 2022; Wang et al., 2022). By contrast, the resilience of students is still an underexplored area. Among the limited number of studies on student resilience, a majority have adopted a quantitative approach based on a range of scales such as the Connor–Davidson Resilience Scale (CD-RISC) (Connor and Jonathan, 2003), the Resilience Scale (Wagnild and Young, 1993), and the scale by Kim and Kim (2017). A common observation from these studies is that resilience has a positive correlation with language competence and second language enjoyment (Wei et al., 2022).

Taken together, the aforecited literature has added to our knowledge of the vital role of resilience in students’ academic performance. Yet, much research is warranted in this under-represented line of inquiry, in particular concerning the specific group of doctoral students. Inspired by these gaps in the literature and the significance of resilience in influencing individuals’ performance and wellbeing, the present research was designed to explore the following two research questions:

1. How do the doctoral students build their resilience during their Ph.D. research?
2. What are the factors influencing doctoral students’ resilience-building process?

## 3 Materials and methods

### 3.1 Participant

In order to provide a rich description of individuals’ lived experiences (Clandinin and Connelly, 2000; Barkhuizen et al., 2014), this research adopted a narrative inquiry approach to explore the resilience-building process of a doctoral student, Hongxia (pseudonym), as she pursued her Ph.D. research at a research-oriented university in the United Kingdom. Before her Ph.D. study, Hongxia worked as a teaching assistant and then as a lecturer at a comprehensive university in China for 7 years. As a university lecturer with a degree of master of arts, she was occasionally warned by her department director and other senior colleagues of the necessity of doing a Ph.D. and receiving a doctoral degree. The university where she worked stipulated that those lecturers who were less than 40 years should hold a doctoral degree to get promoted to an associate professor. “Almost in every weekly meeting, our dean would emphasise this requirement for younger teachers who were in their 30s, including me” (from interview).

Under such circumstances, some of the younger teachers in Hongxia’s department began to pursue their Ph.D. studies in mainland China, Hong Kong, or some other places around the world, such as the United Kingdom, the United States, Australia, Canada, and Malaysia. One of her colleagues, who was also her prior classmate in university, went to do a Ph.D. at a university in Hong Kong. She was also encouraged by this and was thus determined to do a Ph.D. So she began to prepare the application for her Ph.D. study abroad, specifically at a university in the United Kingdom. After considerable effort, she succeeded in the application and was also awarded the joint scholarship provided by the China Scholarship Council and the university in the United Kingdom.

### 3.2 Data collection

The current research and its data collection procedures were approved by and monitored by the institutional ethical review board of the university where she studies. The primary data for this study involved an interview and reflective journals. Specifically, in order to obtain a nuanced understanding of the participant’s resilience-building experiences, a retrospective semi-structured interview was conducted at the end of Hongxia’s Ph.D. research (see Appendix A for the interview guideline). In addition, Hongxia was requested and agreed to write reflective journals to note down what she thought were critical incidents pertinent to her research work or life. When doing this research, the author was also doing a Ph.D. in language and linguistics at the same school as Hongxia. As Hongxia was the researcher’s Ph.D. colleague and they had close contact during daily study and life, the researcher and the participant had built rapport with each other during the research process, thus facilitating the data collection for the present research.

As the researcher and the participant were studying in the same department and had similar research interests, they also had much daily contact with each other. Therefore, the current research was also enlightened by some other informal data. For instance, the researcher and the participant usually had some ordinary conversations in terms of their research as well as participated in many daily activities together. The researcher also noted down some ethnographic observations of the research activities the participant took part in, such as attending some research-related seminars, workshops, or conferences held in and out of the university. In addition, the researcher also collected some other pieces of information related to the participant, such as the WeChat moments the participant posted, which showed her daily thoughts and lives in the forms of texts, pictures, and videos, to inform and triangulate the analysis and findings of the present study.

### 3.3 Data analysis

The data analysis, or the storytelling and re-storytelling process of the present study, was informed by a qualitative thematic analysis (Guest et al., 2012). Meanwhile, the analytical process was also enlightened by the three-dimensional narrative approach (Connelly and Clandinin, 2006), taking account of the dimensions of interaction, continuity, and situation.

**FIGURE 1:**
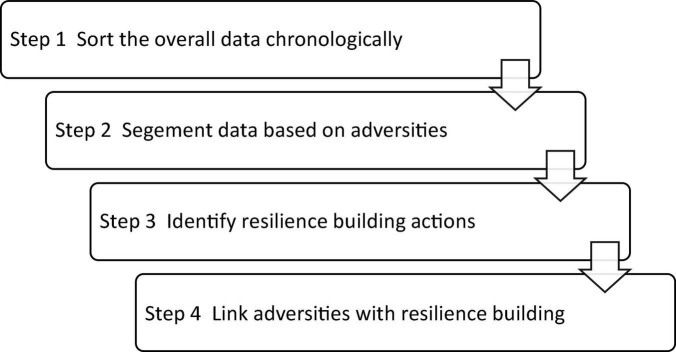
Procedures of data analysis in the present study.

Specifically, the whole process followed the four major procedures, as shown in Figure 1, and the data were first sorted chronologically from the participant’s commencement of her Ph.D. journey to the end of the last year of her Ph.D. research when she submitted her Ph.D. thesis for examination. Second, the data were then segmented according to the adversities she encountered. For example, the difficulties or cheers she experienced during different stages of her Ph.D. research, that is, the first, second, and third years, were identified. Third, the corresponding resilience-building actions for handling the adversities were identified. Finally, the data were analyzed by linking the adversities with resilience-building processes in different stages of her Ph.D. research. By this means, three themes were identified for the participant’s resilience-building process.

Notably, during the analytical process, another researcher was invited to independently code the data and then discuss the disputed parts until a consensus was reached. Furthermore, the analysis and interpretation of the study were also sent to the participant for member checking. These measures were taken to better ensure the rigor and trustworthiness of the study.

## 4 Hongxia’s resilience-building stories

**FIGURE 2:**
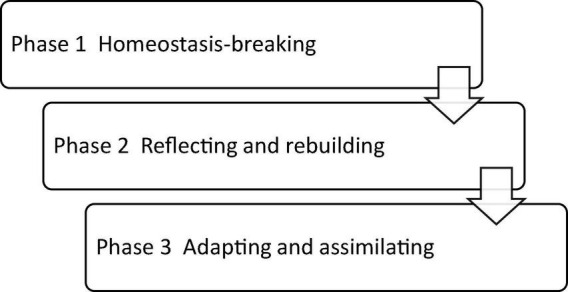
Hongxia’s resilience-building process.

An analysis of the data resulted in three main themes of the resilience-building process by the participant, as shown in Figure 2. The following three narratives describe the challenges that Hongxia had encountered in various stages of her Ph.D. research and the ways in which she was engaged to exercise her agency and build her resilience against those challenges.

### 4.1 Homeostasis-breaking process

As noted earlier, Hongxia had worked at a university in China for 7 years before she commenced her Ph.D. research at a university in the United Kingdom. Thus, she was arguably to be in her mid-career professional life phase based on Gu and Day’s (2007) classification of the three scenarios of teachers’ professional stages. This was also embodied in her personal life. When she began her Ph.D. study, she had got married and had a baby who was then only 10 months old. She had to go to the university by herself and leave her baby at home due to her uncertainties about the upcoming study in a nation where she had never been. This turned out to be very hard, both physically and emotionally, for her as a mother who was used to the days of being together with her family, in particular with the baby. Such emotional challenges were compounded by the seemingly endless days she could then foresee in doing a Ph.D. during the early stages when she arrived at the university in a different country.

When she arrived in the United Kingdom, life was totally different from what she experienced back at home in China. She had undertaken her teaching work and taken care of the baby with the help of her family, which was, in general, with a rather slow pace. However, her life of doing a Ph.D. was in a completely new style, either in daily life or in the research study. This was particularly true in the first year when she was exposed to a new living environment. With the help of a friend, she got the chance to rent and live in a homestay, together with the landlord couple and another undergraduate girl student from the United Kingdom. More importantly, she had to work on her research under the guidance of her supervisors and attend a range of lessons related to her research, such as research methods and second language acquisition. Thus, it was of paramount importance for her to become accustomed to the new study and life habits in a new environment.

Indeed, this sudden change in her lifestyle and environment formed a big shock to her despite that she had some emotional and psychological preparations in advance. In particular, her life seemed to be imbued with study and work at the time. Being far away from home, she felt particularly guilty that she had no opportunity to take care of her child as when she was at home before. Every time when she was tired of the study and needed a break, her feeling of guilty was stronger as she thought she should have spent the time fulfilling her family commitments.

Such a paradoxical feeling increased her emotional pressure and even led to some other new problems in her life. She became very sensitive to the environment around her, which she claimed rarely happened to her before. For example, she felt very disturbed and annoyed by the showering noise made by her flatmate every mid-night, and even could not fall asleep every night until 1 or 2 o’clock after everything was quiet.

The girl took a shower at about twelve or one o’clock every night. And the showering room was just next to my bedroom. It somehow made strange noise every time she took a shower. That really annoyed me and caused increasing tension in my nerves. I tried to tell her but it seemed to be not easy to change as she said that she was used to taking a shower before going to bed. Everyday my nerves were very stressful, increasingly more sensitive to any sound around me. I tried to adjust myself, tried to use ear plug, to listen to music, and to do more exercise during the daytime, but they all seemed to be in vain. So after living there for six months, I left that house and moved to a new place although I could not get my deposit back because I did not live there for one year as initially agreed on with the landlord. (from interview)

In addition, issues pertinent to study and research also posed a big challenge to her. For instance, being used to a daily noon nap at home, Hongxia found it hard to adapt herself to the schedule of the lessons. The classes she attended were often scheduled from 9 or 10 o’clock in the morning to 1 or 2 o’clock in the afternoon, with very limited time intervals at noon for lunch, let alone the nap she usually did when she was at home before. She could not help feeling sleepy every class in the afternoon. “No sleeping at noon ruined the whole afternoon” (from Hongxia’s reflective journal). Furthermore, as she read the literature related to her proposed topic for the Ph.D., she also began to notice that some of the ideas in her proposal might not be feasible enough, and she might need to adjust it for her future research work.

In short, it turned out that the comprehensive issues related to Hongxia’s psychological tension caused by the imbalance between life and work, her inadaptability to the new homestay environment, and the new challenges arising from her study and research work resulted in her inefficiency in work during the daytime. Such inefficient work, in turn, aggravated her feeling of guilt, which seemed to form an incompatible circle. This initial incompatibility in the early stage of her doctoral life represented a homeostasis-breaking process, which featured adversity and disruption in an individual’s resilience-building process (Richardson et al., 1990). In such an adverse situation, various challenging factors, such as stressors and riskers, broke the well-established state of her life. However, this could also serve as a reflecting and motivating factor for agentive individuals to work through the problems and learn new skills from the disruptive experience (Xue, 2021), as noted by Hongxia:

Are there any other things worse than what happened during these days? People say things would get better if it cannot be worse. I do think something needs to be changed and hope it would get improved in the future. (from Hongxia’s reflective journal)

### 4.2 Reflecting and rebuilding

Hongxia endeavored to make every effort to change this situation after she realized the potential danger of this paradoxical status and the necessity of an improvement in her emotional and psychological states. During the daily video call with her family, she sometimes talked about her feelings with her husband. Her husband constantly helped her analyze her condition and encouraged her to learn to relax from the *status quo*. He also suggested some ways of improvement, such as doing more exercise and trying to communicate thoughts with other people. Encouraged by her husband, she began to reflect on the ways of doing her research in an effective and efficient way. She became aware of and came to recognize the necessity of striving for a balance between work and leisure. So she gradually went out of her office and tried to temporarily leave her study to communicate with people around her, such as talking to her friends and Ph.D. colleagues. She expressed the unstinting support of her husband in helping her out of the tensions she experienced, in particular during her Ph.D.:

I felt very hard during the first few months when I came to UK. Homesickness, especially the strong longing for my son, coupled with the difficulties in studying, rendered me to thinking of giving up this study and returning home. I think I was lucky, as during this process, my husband gave me endless and unstinting support. I really couldn’t thank him more. He took care of our son during the night, and went to work during the daytime, from the time when my son was ten months old. About one year after that, he told me that the first night when they slept after I was in UK, he couldn’t help feeling very upset, as he thought that our son was so young that he couldn’t live together with his mom. And my husband was also not sure whether he could take very good care of our baby, such a little baby, you know. But he didn’t tell me this until after almost one year when we all seemed to be more used to this kind of life. He always tells me the good news, encourages me and tries every effort to make me feel relaxed. This is really a great support for me to continue my PhD. (from interview)

As noted earlier, she moved out of the homestay due to the seemingly irreconcilable living habits between her and the flatmate and then rented a new flat together with her friend. This move has, to a large extent, improved her sleeping quality and brought new possibilities to her life. “Things changed a lot after I left the homestay environment, or maybe it is my own mental or psychological problem, I had a very sound sleep every night after moving to the new flat” (from interview). The close contact with her new flatmate also further cheered her up and motivated her to take more exercise. Her new flatmate’s regular swimming exercise also aroused Hongxia’s interest in learning to swim. Then Hongxia and several other friends learned together from her flatmate how to swim. This was not only one skill learning activity but also a social opportunity for Hongxia, which offered considerable help in her effort to strike a balance between work and life.

I had learned many times during the past few years how to swim but did not succeed. So swimming was a big challenge for me. But with my flatmate’s encouragement, I could finally manage it. This was indeed cheerful and fulfilling for me, not just because I learned a new skill, but also enhanced my confidence of working on something I had thought impossible. Since then, I also had a regular exercise activity and sometimes went to swimming by myself. (from interview)

In addition, with the conclusion of the lessons she took after two terms, she could arrange her time for research more flexibly, such as taking a nap during the noon when she wanted to. More importantly, she also gradually developed her thoughts and decided on the specific topic she would do for her Ph.D. research. Her two supervisors are also very supportive, scheduling their meetings regularly to ensure if she was on track. She could also communicate more effectively with her supervisors, knowing what help she could expect and how to ask for feedback from each of them. For example, after knowing Hongxia had difficulty in finding related participants for her Ph.D. research, her supervisors made use of their personal and work relations to help her. Finally, she had enough participants. Also her second supervisor was a psychologist and also the mental health officer of the school. She was good at solving mental problems. So Hongxia turned to her for help when she encountered some disruptive issues, as noted in the following text:

I seemed to be rather seriously disturbed by other people’s messages for requesting me to do something, for example, asking me to translate some abstract for them, to buy something for them when going back home, or even to buy milk for their babies, and so on. If I refused to help them, they would probably be angry with me. However, if I agreed to help them, that would make me feel very distracted and stressful. I was so overwhelmed by these things that I could not concentrate appropriately on my research. I felt greatly relieved after that and also learned how to deal with such a situation. (from interview)

It went without saying that she encountered a number of difficulties and adversities germane to her research. However, she felt that she became gradually accustomed to the environment and learned to resort to the rich resources and affordances the university provided to enrich her knowledge and skills and adjust her psychological state of mind. Through active participation in a variety of activities in addition to doing her research, such as workshops, seminars, forums, or informal discussions with other Ph.D. colleagues or academics, she had been engaged in a dynamic process of reflecting on her research and life and rebuilding her identity and resilience as a Ph.D. student. For example, she attended one workshop related to the life of doing a Ph.D. and was deeply impressed by saying that “Ph.D. is a journey full of tears and cheers”. She felt she could not agree more with this vivid expression. Such a recognition of doctoral students’ normal life, echoed by other Ph.D. colleagues and academics, enhanced her identity and sense of belonging within the research community in which she was embedded, thus contributing to her resilience building during the Ph.D. process.

### 4.3 Adapting and assimilating

Although full of ups and downs, Hongxia’s Ph.D. journey largely proceeded smoothly as scheduled, and she became more adaptive to the research study routine. As she stepped into the third year, Hongxia had collected the required data, made an analysis of them, and moved to the writing-up stage. She could more efficiently manage her time and keep a balance between work and leisure. She could write the thesis as planned and felt much more fulfilled than ever. Her state of mind has had considerable change compared with the initial stage of her Ph.D. She was equipped with more technological skills in handling her data by attending a range of workshops provided by the school and the university. She reported her progress to her supervisors at regular intervals and discussed relevant emerging questions with them. This cheerful experience can be evidenced by one of her WeChat moments, in which she wrote, “The blue and bright sky after meeting with my supervisors” and attached a photo she took when she went out of her supervisor’s office.

Her improved adaptability can also be evidenced by her ways of adjusting herself when confronted with difficulties during this stage of her Ph.D. There was once when she talked with her Ph.D. colleague about their progress of the research. After feeling a little upset about her slow progress compared with the Ph.D. colleague, she composed the following two different versions of episodes (from Hongxia’s reflective journal), which she claimed to be a mini-drama to depict this phenomenon in doctoral students’ life:

**Episode 1**

**Table:** 

A	How is your research going?
B	Ummm: it’s…okay… hmmm slowly… I use a mixed method research. Now I’ve just finished my quantitative data analysis, but haven’t begun the qualitative part yet.
A	(hm good! I am not alone on the slower side) Which year are you now in your Ph.D.?
B	I am approaching the end of my third year.
A	You are doing fine. You know, most Ph.D. students need four years. (Oh, yeah! I’m in the beginning of my second year, and I’ve already completed the quantitative section of the mixed method analysis)

**Episode 2**

**Table:** 

A	How is your research going?
B	Well, it goes very well. I use a mixed method research. Now I’ve just finished my quantitative data analysis, and will begin to do the qualitative part.
A	(Oh, no! I must be the slowest one. I’m so upset) Which year are you now in your Ph.D.?
B	I am in the beginning of my second year.
A	You are going very fast. (Oh, No! I’m approaching the end of my third year of my Ph.D., but I’m still doing the quantitative section of the mixed method analysis)

With the progress in her research, she also became more assimilated into academia. In addition to writing her Ph.D. thesis, Hongxia attended postgraduate forums and conferences to present her research. To her great surprise, her abstract was accepted by the British Association for Applied Linguistics conference. This greatly encouraged her and enhanced her confidence in her research. During the conference presentation, she received many informative questions, comments, some suggestions for her research from Ph.D. colleagues and scholars in other institutions. Her communication with them also motivated her to further reflect on her own research, providing new affordances to her Ph.D. journey. This assimilation culminated in her publication of a research article in the third year of her Ph.D. research.

Furthermore, as she had a rather clear planning for and control of her upcoming life in doing the Ph.D., Hongxia also invited her family to join her. Her husband and kid arrived in the United Kingdom to join her for half a year. During that time period, Hongxia was filled with great happiness and satisfaction with her life.

In the first few days, my son’s jet lag seemed to be completely adjusted. When he woke up in the middle of the night, he always hugged me over and over again and said, “Mom, I like you.” These actions of my son made me feel happy but also a little sad: after over two years’ intermittent separation, how much he missed his mother … After my husband arrived, he did almost all the cooking and other chores, took care of me and my son in every possible way, and gave me great support. (from interview)

Overall, Hongxia’s Ph.D. research and life transited into a steadier stage through her persistent resilience-building process with the help of supervisors, Ph.D. colleagues, academics, and families. Irrespective of ups and downs, she was more adaptive to the doctoral research study and daily routines, considering “tears and cheers” as integrative components of the Ph.D. journey. She established a good relationship with her supervisors and received the support and guidance from them. She became more capable of adjusting her state of mind and handling difficulties and disruptive experiences with an optimistic viewpoint. Her effort in doing the research was echoed and reconfirmed by her Ph.D. colleagues and academics in and out of the university through her participation in various academic activities, such as the aforementioned forums and conferences. Through this means, she felt being assimilated into academia and reconfirmed her identity as an early career scholar in the research community. These relationships, alongside her family support, all contributed to her resilience-building process and her professional identity as a scholar in the making.

## 5 Discussion

The earlier section has outlined the three stages of resilience-building process by the participating Ph.D. student, Hongxia. Throughout the varying stages, some concomitant threads of factors influencing how the participant had gradually built her resilience also emerged from the data analytical process. This section discusses the major representative influencing factors as evidenced by the current research. Taken together, these factors consist of the Ph.D. student’s relationship with the constellations of others and her individual agency.

First, relationships with crucial others in the institutional settings also contributed to the Ph.D. student’s resilience building. In this case, the Ph.D. participant Hongxia moved out of her homestay and gradually made good friends with her peers. She and her friends went out together to learn to swim from her flatmate. This had a positive effect on her resilience-building process. Such friendship from peers assisted the Ph.D. student’s recovery from adversity and adjustment to work and life. More importantly, supervisors are also an instrumental factor in helping the students’ resilience building. The student reported her supportive supervisors in helping her through various difficulties, such as finding appropriate research participants in her data collection process, dealing with psychological problems, and discussing emerging issues related to research. All these supports acted as significant empowerment for the Ph.D. student during the doctorate journey. In addition, the university also provided necessary workshops, seminars, and forums for the Ph.D. student related to her research, which are useful affordances for her resilience building. Such intra-institutional factors, alongside the broader inter-institutional conferences, were conducive to the reflecting, rebuilding, adapting, and assimilating processes of the Ph.D. student and ensured her survival in the demanding journey of a Ph.D. research. They constituted the wider social networks of the Ph.D. student (Cullen et al., 1994; Francis, 2007) and also corroborated existing research on the role of relationships in mediating Ph.D. students’ experience and their transition to independent scholars (Baker and Pifer, 2011; Adams, 2019).

In addition, the family was observed as a critical factor influencing the Ph.D. student’s resilience building. In the initial stage of her Ph.D. in this study, participant Hongxia left her family at home and commenced her Ph.D. research in a study abroad context. This shift in environment and focus on life has caused a considerable shock to her emotional and psychological wellbeing. In particular, the Ph.D. student’s imbalance between life and work, her inadaptability to the new homestay environment, and the new challenges arising from her study and research work caused her paradoxical feeling of guilt and lower efficiency. This represented a homeostasis-breaking stage in her resilience-building process. During this process, her family provided her considerable psychological support, which stimulated and motivated her to make a change to such a status. This indicates the role of family members in Ph.D. students’ resilience building. Indeed, the support of the participant’s family was obvious in every stage of her resilience building. It offered further support to previous research, which argued the significance of work–life balance to the wellbeing of graduate students informed by the work/family border theory (Yusuf et al., 2020).

Furthermore, the Ph.D. student’s resilience building was also influenced by her capabilities to navigate through the anticipated and unanticipated adversities in her professional and personal lives. The stories presented earlier in section “Hongxia’s resilience-building stories” were, in essence, a portrait of how the Ph.D. student Hongxia exercised her agency in the active building of resilience. Across the different stages of her Ph.D. research, she was confronted with various types of difficulties and adversities. Despite the importance of crucial others, individuals’ active response and reaction are of paramount significance in the resilience-building process. Disruptive adversities can also become an opportunity for agentive individuals to work through the problems and learn new skills from the disruptive experience. For example, the imbalance between life and work, her inadaptability to the new homestay environment, the new challenges arising from her study and research work, and her difficulty in soliciting appropriate research participants all stimulated her determination and action in changing her *status quo* by herself or her seeking necessary assistance from crucial others. This echoes the prevalent conceptualization of agency as an intentional act and a socioculturally mediated capacity (Tao and Gao, 2017) and its role in dealing with adverse situations (Gu et al., 2022).

## 6 Conclusion

The present study explored the resilience-building process of a Ph.D. student in a British higher education institution. The analysis identified three stages of the participant’s resilience building: the initial homeostasis-breaking phase, the reflecting and rebuilding phase, and ultimately the adapting and assimilating stage. It also discussed the influencing factors that may impact how the participant built her resilience along with the “cheers and tears” during her candidature. Notably, resilience in doctoral students is not a static or innate nature but is affected dynamically by a range of factors, such as their professional and personal relationships with their peers, supervisors, friends, and families. It is also influenced by their capabilities to navigate through the anticipated and unanticipated adversities in their professional and personal lives.

This research has important implications for researchers, educators, and administrators who are responsible for Ph.D. students’ education and wellbeing in higher education institutions. In light of the earlier findings, an important task for those concerned is not only to have a better understanding of the factors that may influence doctoral students’ resilience over the course of their research but also to sustain these factors that may be conducive to their resilience building in the contexts in which the doctoral students are embedded. Considering these findings, efforts can be made to help doctoral students, from their induction phase, to be aware of the potential adversities and rewards, or the tears and cheers they will likely experience, and the resources, affordances, and means that they can count on to go against the odds and build their resilience. As such, they may be better prepared for the doctoral research journey. As the current research indicated, it can be developed through personal, school, and institutional support, which will ensure that doctoral students sustain their capacity to be resilient and reach their research potential.

As with many other narrative approaches to individual case studies, the present study did not aim to generalize its findings to other cases of contexts but to add to our understanding and knowledge to a particular context, that is, doctoral students’ resilience-building process in this study. Nevertheless, it is also noteworthy that this is merely one doctoral student’s lived experience of resilience building at a particular period of time; therefore, other students or the same student at a different time will likely have different resilience-building experiences. Having said that, the present study has added to the current limited body of research on resilience involved in promoting doctoral students’ wellbeing. The thick description of the resilience-building process by the participant may provide insights for Ph.D. students in similar contexts, no matter which country they may come from. As previously indicated, such individual psychological characteristics are underexplored. Hence, more studies of such lines of inquiry, in particular those involving more participants and longer time duration, are warranted in future.

## Data availability statement

The original contributions presented in this study are included in the article/supplementary material, further inquiries can be directed to the corresponding author.

## Ethics statement

The studies involving human participants were reviewed and approved by the University of Aberdeen. The patients/participants provided their written informed consent to participate in this study.

## Author contributions

The author confirms being the sole contributor of this work and has approved it for publication.

## Appendix A: An interview guideline

1. How was your life prior to the Ph.D. research and study?
2. How do you describe your research and study in different stages of your Ph.D.?
3. How do you describe your life in different stages of your Ph.D.?
4. What difficulties or problems have you encountered during your Ph.D.?
5. How did you go through, handle or solve the difficulties or problems?
6. What cheerful things have you experienced during your Ph.D.?
